# Retraction: Boosting happiness in left-behind children: Unveiling the power of physical activity through cognitive reappraisal and psychological resilience

**DOI:** 10.1371/journal.pone.0342441

**Published:** 2026-02-06

**Authors:** The PLOS One Editors

After publication, the corresponding author requested on behalf of all authors to retract this article [1] due to concerns about the methodology, support for conclusions, and potential for misinterpretation of findings with relevance to policy development. Specifically, they noted a lack of generalizability of the reported results beyond the province of Jiangsu, China, and that failure to adequately measure additional confounding variables, such as duration of parental absence and type of surrogate caregivers, which were not included in the analysis, may significantly bias the results.

Following editorial assessment, PLOS noted that the study was approved by the Ethics Committee of Wuhan Sports University (approval number: 2024029), but the authors are not affiliated with this institution and the data were collected in Suzhou City. The corresponding author stated that permission to recruit participants from schools was also obtained from the Suzhou Municipal Education Bureau. A copy of the ethics approval from Wuhan Sports University was provided for editorial review, but PLOS was unable to reach Wuhan Sports University or Suzhou City University to obtain further information about the ethical oversight procedure.

In light of the concerns raised regarding the study methodology and reliability of the conclusions, the *PLOS One* Editors retract this article [1] in accordance with the request of the corresponding author.

YL did not respond to the final editorial decision. DZ either did not respond directly or could not be reached.
